# Anomalous Levels of CD47/Signal Regulatory Protein Alpha in the Hippocampus Lead to Excess Microglial Engulfment in Mouse Model of Perioperative Neurocognitive Disorders

**DOI:** 10.3389/fnins.2022.788675

**Published:** 2022-03-11

**Authors:** Min Shui, Yi Sun, Dandan Lin, Ziyi Xue, Jianhui Liu, Anshi Wu, Changwei Wei

**Affiliations:** ^1^Department of Anesthesiology, Beijing Chaoyang Hospital, Capital Medical University, Beijing, China; ^2^Department of Anesthesiology, Beijing Shijitan Hospital, Capital Medical University, Beijing, China; ^3^Department of Anesthesiology, School of Medicine, Tongji Hospital, Tongji University, Shanghai, China

**Keywords:** CD47, SIRPα, do not eat me, microglia, phagocytosis, pruning, perioperative neurocognitive disorders

## Abstract

**Background:**

Perioperative neurocognitive disorders (PNDs) are common complications of surgical patients, which can lead to prolonged hospitalization, increased complications, and decreased independence and quality of life. However, the underlying molecular mechanisms of PND remain largely obscure. Microglia activation and synapse loss were observed in PND. Cluster of differentiation 47 (CD47), which can bind to its receptor signal regulatory protein alpha (SIRPα) and generate “do not eat me” signal, protects synapses from excessive pruning. Therefore, we aimed to evaluate the potential role of CD47–SIRPα signaling in PND.

**Methods:**

The tibial fracture surgery was performed in aged C57BL/6 mice for PND model establishment. The expression of CD47 and SIRPα in the hippocampus was assessed. Synaptic plasticity, dendritic spine density, microglial engulfment, and hippocampal-dependent memory function were evaluated after model establishment and intervention with SIRPα overexpression.

**Results:**

CD47 and SIRPα expression in the hippocampus were both decreased after the surgery. SIRPα overexpression showed reduced engulfment within host microglia, but a total effect of excessive synapse engulfment decreased dendritic spine density and post-synaptic density protein 95 (PSD95) expression. SIRPα overexpression could not improve the synaptic dysfunction and cognitive impairment in PND. In addition, SIRPα overexpression led to increased CD47 and Iba1 expression.

**Conclusion:**

Anesthesia and surgery affect CD47–SIRPα signaling. SIRPα overexpression could not ameliorate the cognitive impairment in PND mice. One reason may be that the increased Iba1 expression leads to a total effect of excessive synapse engulfment, which results in decreased dendritic spine density and PSD95 expression.

## Introduction

Perioperative neurocognitive disorders (PNDs) are common complications of surgical patients, especially in elderly patients. The PNDs are characterized by reduction in hippocampus-dependent cognitive functions involving impairment in memory, consciousness, and attention (Evered et al., 2018; Subramaniyan and Terrando, 2019). It also leads to various adverse events, including prolonged hospitalization, increased complications and mortality, and decreased independence and quality of life (Skvarc et al., 2018). Multiple factors were found to be associated with PND. However, the underlying molecular mechanisms remain largely obscure.

Growing evidence has showed that neuroinflammation plays an important role in the pathogenesis of PND (Subramaniyan and Terrando, 2019; Chen et al., 2020). Microglia-mediated neuroinflammation has been linked to PND, as well as other neurodegenerative diseases (Saxena et al., 2021). Microglia, the resident macrophages of the brain, can be activated by different types of stimuli and serve as a surveillance role in eliminating apoptotic debris and invading pathogens. Microglia also play a critical role in phagocytosis and synaptic pruning in the brain, but its activation can also lead to harm (Lehrman et al., 2018; Pluvinage et al., 2019; Werneburg et al., 2020; Ding et al., 2021). Microglia can recognize the “eat me” and “do not eat me” signals to facilitate or suppress the phagocytosis (Lehrman et al., 2018; Rivest, 2018; Pluvinage et al., 2019; Miyanishi et al., 2021). “Eat me” signals have been earlier reported (Hammond et al., 2018; Xiong et al., 2018). In recent years, scientists have paid much attention to negative regulators of phagocytosis—the “do not eat me” signals (Lehrman et al., 2018; Pluvinage et al., 2019; Ding et al., 2021).

Cluster of differentiation 47 (CD47), a membrane protein of the immunoglobulin superfamily, is widely expressed on various cell types, including neurons, microglia, and astrocytes (Oldenborg, 2013; Barclay and Van den Berg, 2014). Signal regulatory protein alpha (SIRPα), a receptor of CD47, is also expressed on microglia and neurons (Gitik et al., 2011; Bedoui et al., 2018; Gheibihayat et al., 2021). CD47–SIRPα interaction can generate “do not eat me” signal, protects synapses from excessive pruning (Lehrman et al., 2018; Ding et al., 2021). In previous studies (Xiong et al., 2018; Liu et al., 2021), microglia activation and synapse loss were observed in PND model. However, the potential role of CD47–SIRPα axis in PND remains unclear.

In the current study, we demonstrated that the protein expression of both CD47 and SIRPα in the hippocampus was significantly reduced in mouse model of PND. Reduced dendritic spine density and increased microglial engulfment are also found in this model. Interestingly, SIRPα overexpression promotes the expression of CD47. However, SIRPα overexpression showed reduced engulfment within host microglia, but a total effect of excessive synapse engulfment decreased dendritic spine density and post-synaptic density protein 95 (PSD95) expression. SIRPα overexpression could not improve the synaptic dysfunction and cognitive impairment in PND. In addition, we found that SIRPα overexpression also led to increased Iba1 expression and an increase in microglia number. That might be the reason for a total effect of excessive synapse engulfment. Together, our data demonstrate the reduced CD47 and SIRPα levels and excessive synaptic engulfment in PND. Too low or too high levels of CD47–SIRPα are detrimental, both can lead to excess microglia-mediated pruning and synapse loss in PND.

## Materials and Methods

### Mice and Treatment

**FIGURE 1:**
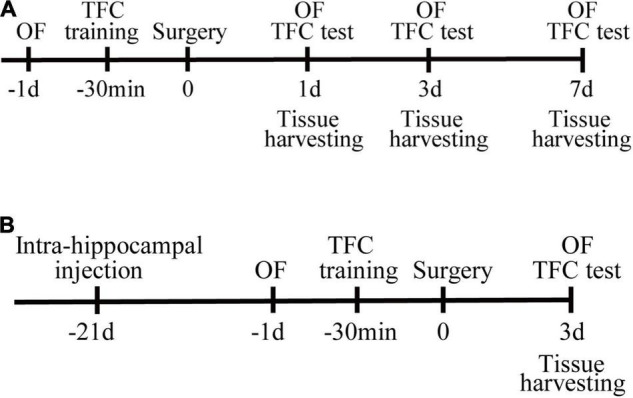
Study design. **(A)** Mice were randomly assigned to control and surgery group. Open-field tests were performed on the day before surgery, postoperative days (PODs) 1, 3, and 7. The training session for trace fear conditioning (TFC) was performed 30 min before surgery. The contextual fear conditioning tests were conducted after the open-field test on PODs 1, 3, and 7. Tissues were harvested at 1, 3, and 7 days after surgery. **(B)** PND mice were randomly assigned to the vehicle or SIRPα overexpression (SIRPα++) group. The SIRPα-overexpressing adeno-associated virus or empty adeno-associated virus vector was injected 3 weeks before surgery. Open-field tests were performed on the day before surgery and POD3. The training session for TFC was performed 30 min before surgery. The contextual test was conducted after the open-field test on POD3. Tissues were harvested on POD3. OF, open field; TFC, trace fear conditioning.

All experiments were approved by the experimental animal ethics committee of Capital Medical University (approval number: AEEI-2020-117) and in accordance with Laboratory Animal Care Guidelines. Male C57BL/6 mice (12–14 months of age) were purchased from the Beijing Weitong Lihua Experimental Animal Technology Co., Ltd. All the mice were kept under standard conditions and had ad libitum access to food and water. The experimental flow chart is illustrated in Figure 1.

### Establishment of the Perioperative Neurocognitive Disorder Mouse Model

The tibial fracture mouse model was established as previously (Xiong et al., 2018). Mice were anaesthetized with 3% isoflurane for induction in a mixture of 100% O_2_. Ropivacaine (0.2%) was administered subcutaneously after anesthesia induction. Then, mice received an open tibia fracture with intramedullary pinning under 2% isoflurane for anesthesia maintenance. The skin was closed with interrupted stitches (4-0 silk suture). The procedure lasted 15 to 20 min. Throughout the surgical and postoperative periods, body temperature of the mice was maintained at 37°C using a heating blanket. Control mice were not subjected to anesthesia or surgical procedure.

### Stereotactic Injection

Intra-hippocampal injections were performed as described previously (Reverchon et al., 2020; Liu et al., 2021). The injection coordinates were 2.3 mm posterior to the bregma, 1.8 mm lateral to the midline, and 2.0 mm ventral to the surface of the dura mater. The overexpression of SIRPα was obtained through local injection of the adeno-associated viral construct AAV.CAG.mSIRPα-3FLAG-P2A-EGFP.WPRE.SV40pA (AAV9 serotype; Beijing Xibei Hongcheng Biotechnology Co., Ltd., China). The adeno-associated viral vectors (AAV9.CAG-EGFP.WPRE.SV40pA) with similar concentrations were injected as control. A two microliters of virus was injected into each side at a rate of 0.25 μl/min. After injection, the microinjection needle was left in place for 5 min to avoid reflux and slowly withdrawn. Three weeks after injection, the mice received an open tibia fracture with intramedullary pinning. Then, mice were euthanized for further analysis.

### Open-Field Test

Exploration, anxiety, and locomotor activity were studied in the open-field test. Mice acclimated to the experimental environment for 30 min before testing. The apparatus for the open-field test was a box (50 × 50 cm). The central area was defined as the center 50% of the apparatus and the remaining as periphery area. The mice were placed into the box from the same location. Before testing, mice were acclimated to the apparatus for 5 min. The total distance, the distance in center zone, and the time spent in the center were recorded for 5 min using Anymaze software (Stoeltus). Alcohol (70%) was used to remove odors in the open-field test box between all sessions.

### Trace Fear Conditioning

Trace fear conditioning has been used to assess hippocampal-dependent memory in rodents as previously described (Feng et al., 2017; Hu et al., 2018). The experiment included two phases: training and test. During the training, mice were allowed to explore the environment for 3 min. Then, they were presented with a conditional stimulus and an auditory cue (75–80 dB, 5 kHz) for 20 s. After 10-s intervals, a 2-s foot shock of 0.7 mA (unconditional stimulus) was administered. The tone and foot shock pairing were repeated with a 60-s interval. Mice were removed from the chamber after an additional 30 s. Surgery was performed within 30 min after training. One, 3, or 7 days later, the mice were tested in the same chamber but with neither tone nor shock. Memory for the context was assessed by freezing behavior during the 5-min test time. The freezing behavior was expressed as a percentage of the observation period and was automatically recorded using Anymaze software (Stoeltus). A decrease in the percentage of freezing time indicated memory impairment.

### Western Blotting

The hippocampal tissue was rapidly separated from the brain on ice. Then, the hippocampus was lysed on ice for 30 min by radioimmunoprecipitation (RIPA) lysis buffer (Beyotime, Jiangsu, China) with a cocktail of protease inhibitors and phosphatase inhibitors (Roche, Manheim, Germany). Protein concentration was quantified by bicinchoninic acid (BCA) protein assay (Thermo Fisher Scientific, Rockford, IL, United States). The protein samples (10 μg for SIRPα and PSD95 and 15 μg for CD47) were subjected to 10% SDS-polyacrylamide gel electrophoresis (SDS-PAGE) gel and then transferred to polyvinylidene difluoride membranes (Millipore, Billerica, MA, United States). Membranes were blocked with 5% skim milk (Cell Signaling Technology, Beverly, MA, United States) for 1 h at room temperature and then incubated with primary antibodies overnight at 4°C. Primary antibodies were as follows: rabbit anti-SIRPα (1:1,500; Abcam, ab8120), rabbit anti-CD47 (1:1,500; Abcam, ab175388), rabbit anti-PSD95 (1:1,000; Cell Signaling Technology, #3450), and mouse anti-Beta Actin (1:3,000; ProteinTech, 66009-1-lg). After washing, the membranes were stained with fluorescent secondary antibodies for 1 h at room temperature. Secondary antibodies were as follows: anti-rabbit IgG (H+L) (Dylight 800 4 × PEG conjugate) (1:15,000; Cell Signaling Technology, #5151) and anti-mouse IgG (H+L) (Dylight 680 conjugate) (1:7,500; Cell Signaling Technology, #5470). The Odyssey CLX Infrared Imaging System (LICOR Odyssey, United States) was used to acquire images.

### Immunohistochemistry

Brains were harvested from mice following transcardial perfusion with precooled normal saline and 4% paraformaldehyde (PFA). The brains were post-fixed with 4% PFA for 4 h at 4°C and then dehydrated in 15% and 30% sucrose for cryoprotection at 4°C. Coronal brain sections of 20 or 40 μm thickness were cut in a Leica cryostat microtome (Leica CM1950). Brain sections were blocked with QuickBlockTM blocking buffer for immunol staining (Beyotime, Jiangsu, China, P0260) for 15 min at room temperature. Afterward, the brain sections were incubated with primary antibodies at 4°C overnight. After washing three times with PBS, brain sections were incubated with fluorescent-labeled secondary antibodies for 1 h at room temperature. Finally, the sections were washed and mounted in 4’,6-diamidino-2-phenylindole (DAPI)-containing mounting medium (ZLI-9557, Zhongshan Golden Bridge Biotechnology, Beijing, China). Images were captured using Leica TCS SP8 X confocal microscope (Leica, Wetzlar, Germany). Quantitative analyses were performed using Fiji software.

Information of antibodies were as follows: primary antibodies: rabbit anti-CD47 (1:100; Abcam, ab175388); rat anti-CD68 (1:100; Abcam, ab53444); rabbit anti-SIRPα (1:100; Abcam, ab8120); rabbit anti-PSD95 (1:100; Cell Signaling Technology, #3450); mouse anti-PSD95 (1:100; Cell Signaling Technology, #36233); rabbit anti-Iba1 (1:100; ProteinTech, 10904-1-AP); and goat anti-Iba1 (1:50; Novus Biologicals, NB100-1028SS); secondary antibodies: Alexa Fluor 488–conjugated goat anti-rabbit IgG (H+L) (1:100; Zhongshan Golden Bridge Biotechnology, ZF-0511); donkey anti-mouse IgG (H+L), CoraLite594 conjugate (1:100; ProteinTech, SA-00013-7); goat anti-rat IgG (H+L) cross-adsorbed secondary antibody, Alexa Fluor 594 (1:1,000; Thermo Fisher Scientific, A-11007); and donkey anti-goat IgG (H+L) cross-adsorbed secondary antibody, Alexa Fluor 633 (1:1,000; Thermo Fisher Scientific, A-21082).

### Three-Dimensional Reconstruction of Microglia and Engulfment Analysis

Brain sections were imaged with the 63× oil immersion objective and 2× electronic zoom using Leica TCS SP8 X confocal microscope (Leica, Wetzlar, Germany) with 0.2-μm z-step. Deconvolution was performed before three-dimensional (3D) reconstruction using Huygens Professional software (SVI, Scientific Volume Imaging, Hilversum, Netherlands). To quantify microglial engulfment of PSD95, 3D image rendering of z-stack images from confocal microscope using Imaris software (version 9.5.0, Bitplane, Switzerland) is based on the protocol from Schafer et al. (2014). The volume of the microglia and PSD95 was calculated using surface rendered images. Engulfment percentage was calculated as volume of internalized PSD95/volume of microglial cell. Microglia and PSD95 3D surface rendering were created separately with a threshold.

### Golgi Staining

Golgi staining was performed according to the manufacturer’s instructions of a FD Rapid Golgi staining kit (PK401, FD Neuro Technologies, United States). Mouse brains were harvested and immersed in the mixture of solution A and B (solution A:solution B = 1:1) for 2 weeks at room temperature in the dark. Then, the brain transferred to solution C for 4 days. The mixture and solution C were renewed within the first 24 h. Coronal brain sections (100 μm thick) of hippocampus were cut using a cryostat microtome (Leica, Wetzlar, Germany). The sections were further stained according to the manufacturer’s instructions. The stained slides were scanned using the Pannoramic scan digital slice scanner (3DHISTECH Ltd., Budapest, Hungary). The images were analyzed by ImageJ software. Secondary and third dendrites were sampled for spine density quantification.

### Electrophysiology

Mice were anesthetized with pentobarbital sodium. The brains were harvested following transcardial perfusion with ice-chilled N-methyl-D-glucamine (NMDG)–based artificial cerebrospinal fluid (ACSF) (92 mM NMDG, 2.5 mM KCl, 1.25 mM NaH_2_PO_4_, 30 mM NaHCO_3_, 20 mM N-2-hydroxyethylpiperazine-N-2-ethane sulfonic acid (HEPES), 25 mM glucose, 2 mM thiourea, 5 mM Na-ascorbate, 3 mM Na-pyruvate, 0.5 mM CaCl_2_, 10 mM MgSO_4_, pH 7.3–7.4, with 95% O_2_ and 5% CO_2_). Then, brains were quickly removed and placed in the same ice-chilled ACSF with 95% O_2_ and 5% CO_2_. The coronal slices (400 μm) containing hippocampus were cut with a vibratome (VT1200, Leica) and sequentially incubated in NMDG-based ACSF (at 34 °C for 12 min) and holding ACSF (containing 92 mM NaCl, 2.5 mM KCl, 1.25 mM NaH_2_PO_4_, 30 mM NaHCO_3_, 20 mM HEPES, 25 mM glucose, 2 mM thiourea, 5 mM Na-ascorbate, 3 mM Na-pyruvate, 2 mM CaCl_2_, 2 mM MgSO_4_, pH 7.3–7.4, with 95% O_2_ and 5% CO_2_) for at least 1 h at room temperature. After that, the slices were transferred to a recording chamber and continuously superfused with recording ACSF (containing 119 mM NaCl, 2.5 mM KCl, 1.25 mM NaH_2_PO_4_, 24 mM NaHCO_3_, 12.5 mM glucose, 1.3 mM Na-ascorbate, 0.6 mM Na-pyruvate, 2 mM CaCl_2_, 2 mM MgSO_4_, pH 7.3–7.4, with 95% O_2_ and 5% CO_2_) throughout the experiments.

Med64 system was used to record field excitatory postsynaptic potential (fEPSP) in the hippocampal slices as previously described (Zhang et al., 2017). We recorded fEPSP with a glass microelectrode (3–5 MΩ, filled with recording ASCF). The hippocampal CA1 neurons were visualized through Olympus microscope (Olympus, Tokyo, Japan) during recording. The stimulus intensity, measurement of the input–output relationship, and long-term potentiation (LTP) induction were performed as described by Zhang et al. (2017). The slopes and peak amplitudes of fEPSP were calculated and recorded. Before LTP induction, baseline responses were recorded for at least 20 min. We recorded for 60 min after LTP induction. The data were presented as the percentages of average values of 10 min before LTP induction.

### Statistical Analysis

Statistical analyses were performed with GraphPad Prism (version 8.0.2). Appropriate tests were carried out to confirm the normality of the data. Comparisons between two groups were applied using unpaired two-tailed *t*-test, when data were conformed to normal distribution. Otherwise, non-parametric tests were used. One-way ANOVA was used for multiple comparisons. Statistical significance was set at *p* < 0.05.

## Results

### Reduced Synaptic Transmission and Dendritic Spine Density in Perioperative Neurocognitive Disorder Model

**FIGURE 2:**
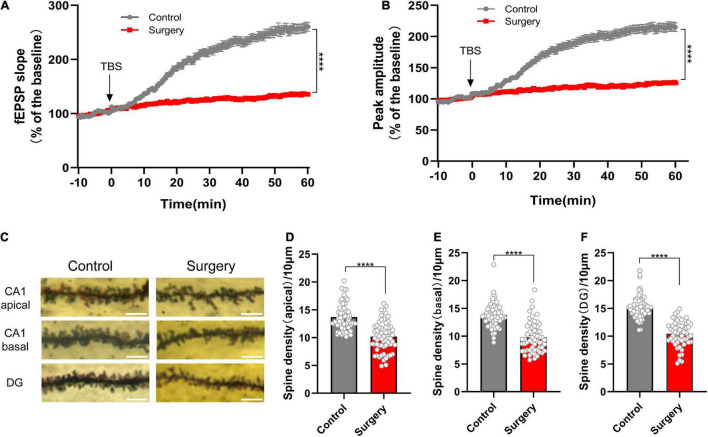
Reduced synaptic transmission and dendritic spine density in PND. **(A)** The slope and **(B)** peak-to-peak amplitude of fEPSP 60 min after TBS, normalized by the averaged recording before TBS (the baseline) in the hippocampal CA1 region. *n* = 5 mice per group, five brain slices per mouse; the last 10 min of the recording were for statistical analysis. **(C)** Representative images of the Golgi-stained dendrites in CA1 and DG neurons from the control and surgery group. Scale bar = 5 μm. **(D–F)** Quantitation of dendritic spine density. *n* = 4 mice per group, 10–13 spines per mice for each region. Tissues for electrophysiology and Golgi staining were harvested on POD3. Data are shown as mean ± SEM. ****p < 0.0001. fEPSP, field excitatory postsynaptic potential; POD, postoperative day; TBS, theta-burst stimulation.

In our previous study, we found that cognitive impairment in PND mice was related to synapsin and PSD95 reduction (Xiong et al., 2018). In the present study, after the PND model successfully established (Supplementary Figures 1A–E), we performed electrophysiology at postoperative day 3 (POD3, according to the time point in our previous study) to record fEPSP. Theta-burst stimulation (TBS) was used for LTP induction. The results showed significant decrease in fEPSP slope and peak-to-peak amplitude after TBS in surgery group (Figures 2A,B). The induction of LTP involves both presynaptic and postsynaptic responses to the stimulation. Expression of LTP refers to the synapse-specific and enhancement of synaptic transmission (Larson and Munkácsy, 2015). It means that anesthesia and surgery affect synaptic transmission. Because of its significance for learning and memory, this result also reflects the success of the modeling. Next, we performed Golgi staining and quantified dendritic spine density. We found that anesthesia and surgery induced spine loss of neurons in CA1 region (control vs. surgery: apical, *p* < 0.0001; basal, *p* < 0.0001; Figures 2C–E) and dentate gyrus (DG) (control vs. surgery: *p* < 0.0001; Figures 2C,F) of hippocampus. Together, we point that the mechanism of PND is largely associated with synaptic dysfunction.

### Orthopedic Surgery Induces Increased Engulfment of Synaptic Structure and CD47– Signal Regulatory Protein Alpha Downregulation in the Hippocampus

**FIGURE 3:**
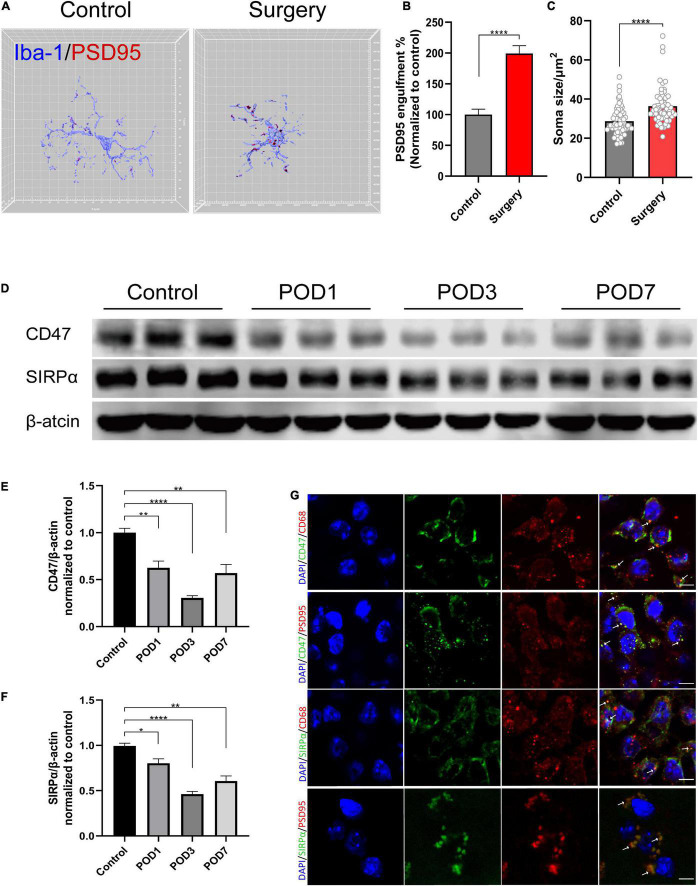
Anesthesia and orthopedic surgery induce increased engulfment of synaptic structure and CD47–SIRPα downregulation in the hippocampus. **(A)** Representative 3D reconstruction and surface rendering of PSD95 inside Iba1^+^ microglia from control group versus surgery group. Microglia in CA1 region were chosen. **(B)** Quantification of PSD95 engulfment within microglia. *n* = 5 mice per group, 8–10 cells per mouse were quantified. **(C)** Quantification of microglial soma size. *n* = 5 mice per group, 11–13 cells per mouse were quantified. **(D)** Representative Western blots of CD47 and SIRPα on postoperative days 1, 3, and 7. **(E,F)** Quantitative analysis of CD47 and SIRPα levels compared with the control group. **(G)** Representative images of immunofluorescence colocalization of CD47 and SIRPα with microglia (CD68) and synapse (PSD95), respectively. White arrows indicate the colocalization. Scale bar, 5μm. Control mice were used in this assay. Data are shown as mean ± SEM. *p < 0.05; **p < 0.01; and ****p < 0.0001. POD, postoperative day.

To further investigate the reason of synaptic dysfunction in PND, we examined synapse engulfment by calculating percent engulfment (volume of postsynaptic marker PSD95/volume of the microglia). Three-dimensional surface reconstruction and rendering showed that the volume of PSD95 within microglia was significantly increased after anesthesia and surgery (control vs. surgery, *p* < 0.0001; Figures 3A,B). The quantification of microglial soma size also showed increased soma size in surgery group (control vs. surgery, *p* < 0.0001; Figures 3A,C). It exhibited increased microglia activation.

CD47–SIRPα signaling acts as “do not eat me” signal to inhibit phagocytosis. Its role in PND is still unclear. According to their cellular localization, we found that CD47 and SIRPα were both colocalized with microglia (CD68) and synapse (PSD95) (Figure 3G). Then, we assessed CD47 and SIRPα protein expression levels in the hippocampus at 1, 3, and 7 days after the surgery, respectively. Compared to the control group, the protein level of both CD47 and SIRPα decreased at 1, 3, and 7 days after the surgery (CD47: control vs. POD1, *p* = 0.008; control vs. POD3, *p* < 0.0001; control vs. POD7, *p* = 0.015; SIRPα: control vs. POD1, *p* = 0.033; control vs. POD3, *p* < 0.0001; control vs. POD7, *p* = 0.002; Figures 3D–F). The decrease of protein level on POD3 was the most significant. Therefore, we chose model of POD3 in subsequent experiments.

### Signal Regulatory Protein Alpha-CD47 Signaling Regulates Microglial Synapse Engulfment

**FIGURE 4:**
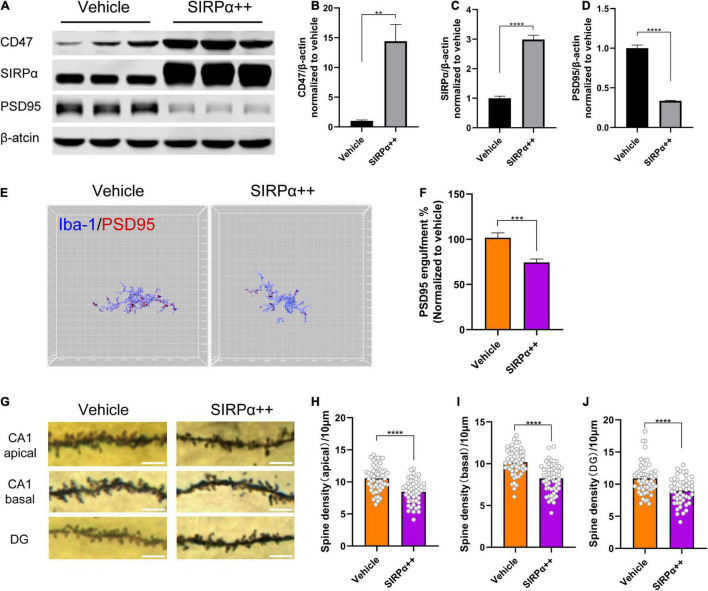
SIRPα overexpression reduces PSD95 engulfment within host microglia but leads to synapse loss. **(A)** Representative Western blots of CD47, SIRPα, and PSD95 on POD3. **(B–D)** Quantitative analysis of CD47, SIRPα, and PSD95 levels compared with the vehicle group. **(E)** Representative 3D reconstruction and surface rendering of PSD95 inside Iba1^+^microglia from SIRPα overexpression mice versus vehicle mice. Microglia in CA1 region were chosen. **(F)** Quantification of PSD95 engulfment within microglia. *n* = 5 mice per group, 8–10 cells per mouse were quantified. **(G)** Representative images of the Golgi-stained dendrites in CA1 and DG neurons. Scale bar = 5 μm. **(H–J)** Quantitation of dendritic spine density. *n* = 4 mice per group, 13–15 spines per mice for each region. Data are shown as mean ± SEM. **p < 0.01; ***p < 0.001; and ****p < 0.0001.

To validate the impact of CD47–SIRPα signaling on microglia engulfment, we overexpressed SIRPα and confirmed the overexpression efficiency (vehicle vs. SIRPα++, *p* < 0.0001; Figures 4A,C). Interestingly, we found that SIRPα overexpression led to increased CD47 expression (vehicle vs. SIRPα++, *p* = 0.0008; Figures 4A,B). This means that CD47 expression is associated with increased expression of SIRPα. The engulfment analysis showed that SIRPα overexpression did reduce the PSD95 engulfment within host microglia (vehicle vs. SIRPα++, *p* = 0.0008; Figures 4E,F). These data suggest that microglial synapse engulfment is regulated by CD47–SIRPα signaling.

### Signal Regulatory Protein Alpha Overexpression Leads to Synapse Loss and Does Not Improve the Synaptic Function and Cognitive Impairment in Perioperative Neurocognitive Disorder Mice

To validate that the surgery-induced synapse loss is due to microglial synaptic engulfment regulated by CD47–SIRPα signaling, we examined dendritic spine density in SIRPα-overexpressing PND mice. Unexpectedly, decreased spine density was found in hippocampus of SIRPα-overexpressing PND mice (vehicle vs. SIRPα++: CA1 apical, *p* < 0.0001; CA1 basal, *p* < 0.0001; DG, *p* < 0.0001; Figures 4G–J). To verify this result again, we assessed PSD95 protein level in SIRPα overexpression group. PSD95 was decreased after SIRPα overexpression (vehicle vs. SIRPα++, *p* < 0.0001; Figures 4A,D). This is in accordance with the decreased spine density.

**FIGURE 5:**
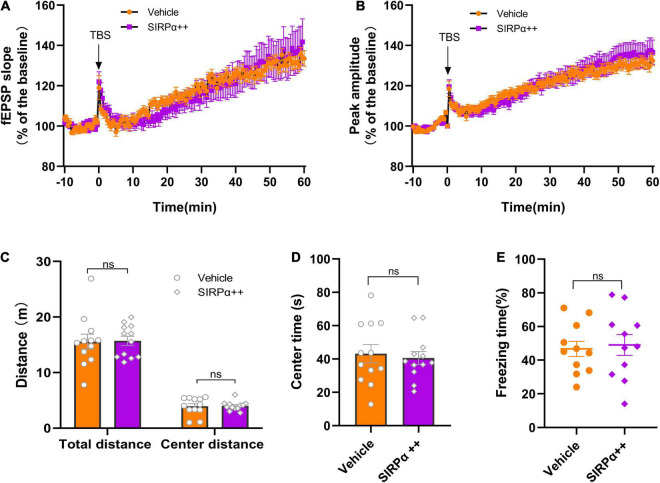
SIRPα overexpression in PND mice cannot improve synaptic function and cognitive impairment. **(A)** The slope and **(B)** peak-to-peak amplitude of fEPSP 60 min after TBS, normalized by the averaged recording before TBS (the baseline) in the hippocampal CA1 region. *n* = 5 mice per group, 5 brain slices per mouse; the last 10 min of the recording were for statistical analysis. **(C,D)** The total distance traveled, center distance traveled, and time spent in the center during open-field test before surgery. *n* = 12 mice per group. **(E)** The percentage of freezing time during the contextual test on POD3. *n* = 12 mice per group. Data are shown as mean ± SEM. ns, not significant. fEPSP, field excitatory postsynaptic potential; TBS, theta-burst stimulation.

Then, we performed electrophysiology and behavioral assays. Electrophysiological results showed no significant difference between vehicle group and SIRPα overexpression group in fEPSP slope and peak-to-peak amplitude (Figures 5A,B). It suggests that there was no improvement in synaptic function after intervention. Open-field tests showed no significant difference between the two groups before surgery (Figures 5C,D). After surgery but before the contextual fear conditioning test, there was also no difference in open-field tests (Supplementary Figures 1F,G). These indicated that the two groups were comparable. Trace fear conditioning also showed no significant difference between the two groups in freezing time (Figure 5E). It meant that hippocampus-dependent memory in both groups was no statistically significant difference. This was consistent with the electrophysiological results. These data demonstrate that SIRPα overexpression does not significantly improve the synaptic function and cognitive impairment in PND.

### Signal Regulatory Protein Alpha Overexpression May Trigger Compensatory Mechanisms of Microglial Engulfment

**FIGURE 6:**
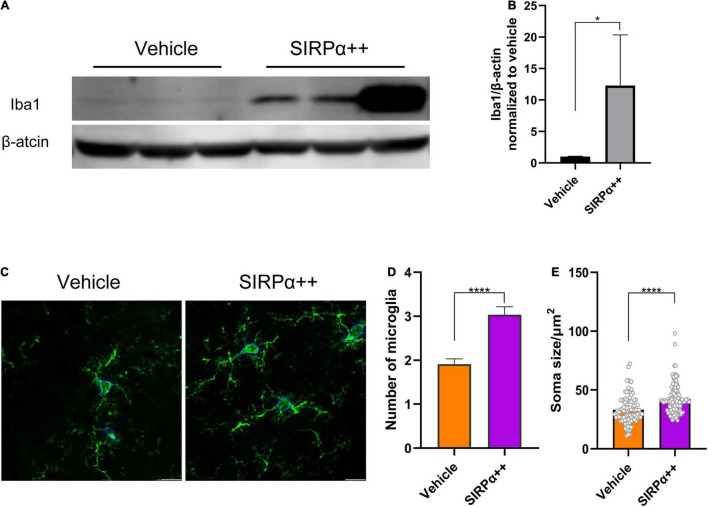
SIRPα overexpression in PND mice leads to increase of microglial activation. **(A)** Representative Western blots of microglial marker Iba1. **(B)** Quantification of Iba1. **(C)** Representative confocal images of microglia in hippocampus and showing measurement of soma size (outlined by blue line). Scale bar = 10μm; the quantified area was 92.46 × 92.46 μm. **(D)** Counts of microglia. *n* = 5 mice per group, average of 11–12 fields from each mouse. **(E)** Quantification of soma size. *n* = 5 mice per group, average of 18–20 cells from each mouse. Data are shown as mean ± SEM. *p < 0.05 and ****p < 0.0001.

We tried to find the reason why SIRPα overexpression could not improve the synaptic function and cognitive impairment in PND model. We assessed microglial marker Iba1 protein level. It showed that Iba1 was significantly increased after SIRPα overexpression (vehicle vs. SIRPα++, *p* = 0.0286; Figures 6A,B). We also counted the number of microglia in visual field at × 63 magnification with a 2× digital zoom in (confocal pictures were used for 3D reconstruction). Compared to vehicle group, the number of microglia in SIRPα overexpression group was increased (vehicle vs. SIRPα++, *p* < 0.0001; Figures 6C,D). The activated microglia increase the size of their body. We quantified the soma size of microglia in both groups. It showed that the average soma size of microglia in SIRPα overexpression group was also increased (vehicle vs. SIRPα++, *p* < 0.0001; Figures 6C,E). It meant that activated microglia in SIRPα overexpression group were more than vehicle group. These results suggest that SIRPα overexpression may trigger compensatory mechanisms of microglial engulfment.

## Discussion

It has been found that CD47–SIRPα signaling could protect synapse from excess microglia-mediated phagocytosis in developing retinogeniculate system (Lehrman et al., 2018), as well as in model of Alzheimer’s disease (Ding et al., 2021). In this study, we found that the protein level of CD47 and SIRPα were both decreased after anesthesia and surgery. At the same time, decreased dendritic spine density and increased microglial synaptic engulfment were found in PND model. Previously, we found that C3/C3aR (an “eat me” signal) could regulate microglial phagocytic activity and administration of C3aR antagonist could alleviate surgery-induced synapse loss and cognitive impairment in PND mice (Xiong et al., 2018). It suggested that synapse loss and cognitive impairment in PND were associated with microglia activation and engulfment.

We hoped to verify that we could prevent excessive engulfment and rescue synapse loss through upregulation of SIRPα. We found that CD47 expression was upregulated after overexpression of SIRPα. CD47 is a ligand of SIRPα, and CD47–SIRPα interaction acts as a “do not eat me” signal to inhibit phagocytosis. It has been reported that cytokines (e.g., tumor necrosis factor alpha, interferon-γ, and interleukin-6), microRNAs, and enzymes can regulate the expression of CD47 through various pathways (Huang et al., 2020). However, the mechanism of SIRPα-induced CD47 upregulation is not clear. Further investigation may be required.

We successfully overexpressed SIRPα. In addition, we found that the average engulfment within host microglial cell was reduced. This verifies that CD47–SIRPα signaling negatively regulates microglial phagocytosis. However, dendritic spine density and PSD95 expression were both decreased in SIRPα overexpression group, which meant that SIRPα overexpression led to more synapse loss. Absolutely, cognitive impairment could not be improved by SIRPα overexpression. This is verified by behavioral test and electrophysiological results. These results may have emerged for several reasons.

Han et al. (2012) demonstrated that modulating CD47 function at different stage had opposing effects in the peripheral immune system and the central nervous system. Similarly, too low or too high level of CD47 (or SIRPα) may have harmful effect, resulting in excess engulfment and synapse loss. However, we could not regulate the extent of SIRPα overexpression to the normal level. Secondly, CD47–SIRPα interaction enables the docking and the recruitment of Src homology region 2 domain-containing phosphatase–1 (SHP-1) and SHP-2 (Gheibihayat et al., 2021). Studies have demonstrated that SHP-1 and SHP-2 perform opposite biological functions (Neel et al., 2003). SHP-1 negatively regulates cell functions, such as phagocytosis (Ohnishi et al., 2005). Conversely, cell activity like growth and migration has been reported to be enhanced by SHP-2 (Gheibihayat et al., 2021). It also has been reported that SIRPα can promote NO production via JAK2/STAT pathway (Alblas et al., 2005). What is more, many other components [such as c-Src tyrosine kinases (CSK), proline-rich tyrosine kinase 2 (PYK2), and the adaptor molecules growth factor receptor-bound protein 2 (Grb2)] can bind to SIRPα, to regulate corresponding signaling (van Beek et al., 2005). Therefore, SIRPα overexpression might modulate other cell functions. Theoretically, possible explanations can account for these results.

SIRPα overexpression led to decreased PSD95 expression and dendritic spine density, but without worse cognitive impairment, which may also due to complex body regulation and downstream mechanisms of CD47–SIRPα signal. What is more, microglial phagocytosis is only a part of the pathogenesis of neurocognitive impairment, and there may be other possible mechanisms and directions.

We also tried to find some experimental support. The quantitative analysis of the fluorescent images showed increased number of microglia. In addition, Western blot result showed that Iba1 level was increased in SIRPα overexpression group. Combined with decreased dendritic spine density and PSD95 expression, we speculate that, although engulfment of host cell was reduced via SIRPα overexpression, the total effect of phagocytosis was increased. This may be the explanation for no significant improvement in behavioral test and electrophysiological results.

There are some limitations in this study. We did not find effective agonist for CD47 or SIRPα. In addition, the feasibility and efficiency of using gene knockout mice were low in our study, because aged mice were required in this model. Therefore, we had to choose adeno-associated virus (AAV) to overexpress SIRPα. In addition, the efficiency of AAV transduction in microglia is limited (Maes et al., 2019), which may affect the result, although we found reduced expression of CD47 and SIRPα and increased synaptic engulfment, synapse loss, and cognitive impairment in PND model. We also found that CD47–SIRPα signaling inhibited host microglial engulfment. However, because of the complexity of research *in vivo*, we did not find direct evidence to suggest that we could regulate microglial engulfment to reduce synapse loss and improve cognitive impairment in PND model through modulating the expression of SIRPα. Although there was no direct evidence to support a complete signaling pathway, there were also several novel findings in this study. We hope to explore the mechanism of their interaction in further study.

## Conclusion

In summary, excess microglial synaptic engulfment is associated with PND. Anesthesia and surgery affect CD47–SIRPα signaling. In addition, CD47–SIRPα signaling regulates phagocytosis of host microglia in PND. SIRPα overexpression could not ameliorate the cognitive impairment in PND mice. One reason may be that the increased Iba1 expression leads to a total effect of excessive synapse engulfment, which results in decreased dendritic spine density and PSD95 expression. Further study is needed to find a novel therapeutic target to regulate microglial engulfment in PND.

## Data Availability Statement

The original contributions presented in the study are included in the article/Supplementary Material, further inquiries can be directed to the corresponding authors.

## Ethics Statement

The animal study was reviewed and approved by the Experimental Animal Ethics Committee of Capital Medical University (approval number: AEEI-2020-117).

## Author Contributions

CW and MS designed the research. MS, DL, and ZX performed the research. MS and YS analyzed the data. MS wrote the original draft preparation. JL, AW, and CW wrote, reviewed, and edited the manuscript. AW and CW supervised the manuscript and acquired the funding. All authors approved the final manuscript.

## Conflict of Interest

The authors declare that the research was conducted in the absence of any commercial or financial relationships that could be construed as a potential conflict of interest.

## Publisher’s Note

All claims expressed in this article are solely those of the authors and do not necessarily represent those of their affiliated organizations, or those of the publisher, the editors and the reviewers. Any product that may be evaluated in this article, or claim that may be made by its manufacturer, is not guaranteed or endorsed by the publisher.

## Supplementary Material

Supplementary Figure 1 Behavioral tests. **(A,B)** The total distance traveled, center distance traveled, and time spent in the center during open-field test before surgery. *n* = 13 mice for control group, and *n* = 15 mice for surgery group. **(C,D)** The total distance traveled, center distance, traveled and time spent in the center during open-field test before the contextual fear conditioning test. *n* = 13 mice for control group, and *n* = 15 mice for surgery group. **(E)** The percentage of freezing time during the contextual fear conditioning test on POD3. *n* = 10 mice per group. **(F,G)** The total distance traveled, center distance traveled, and time spent in the center during open-field test before the contextual fear conditioning test. *n* = 12 mice per group. Data are shown as mean ± SEM. ns, not significant; ^∗∗^*p* < 0.01.
