# Supplementary figures and images for: Anomalous Levels of CD47/Signal Regulatory Protein Alpha in the Hippocampus Lead to Excess Microglial Engulfment in Mouse Model of Perioperative Neurocognitive Disorders
Source: Front Neurosci. 2022 Mar 11;16:788675. doi: 10.3389/fnins.2022.788675 (PMC8962642; doi:10.3389/fnins.2022.788675)

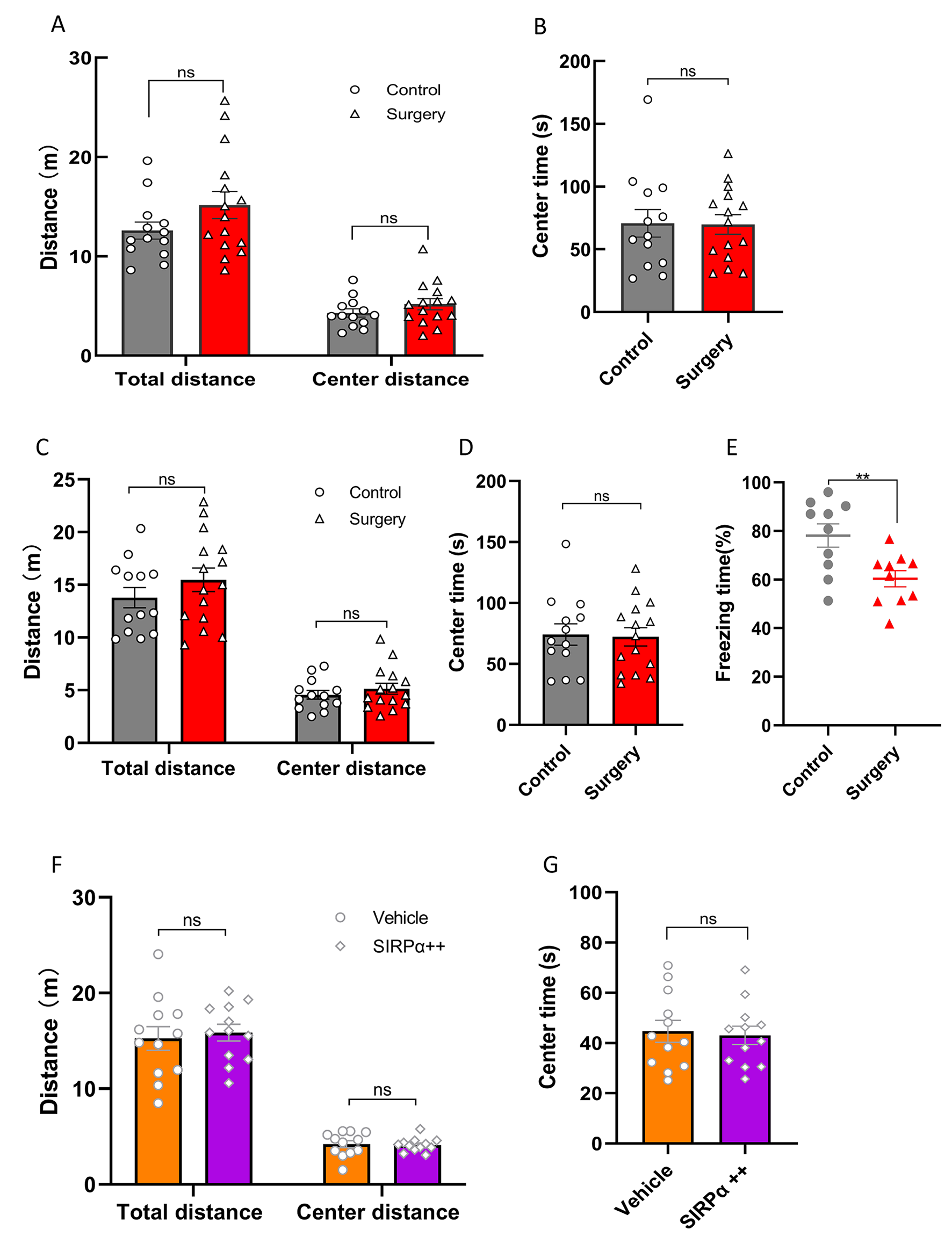

Supplement: Supplementary Figure 1 — Behavioral tests. (A,B) The total distance traveled, center distance traveled, and time spent in the center during open-field test before surgery. n = 13 mice for control group, and n = 15 mice for surgery group. (C,D) The total distance traveled, center distance, traveled and time spent in the center during open-field test before the contextual fear conditioning test. n = 13 mice for control group, and n = 15 mice for surgery group. (E) The percentage of freezing time during the contextual fear conditioning test on POD3. n = 10 mice per group. (F,G) The total distance traveled, center distance traveled, and time spent in the center during open-field test before the contextual fear conditioning test. n = 12 mice per group. Data are shown as mean ± SEM. ns, not significant; ∗∗p < 0.01. [file Image_1.TIF]
